# Interventional Versus Conservative Strategy in Patients With Spontaneous Coronary Artery Dissections: Insights From DISCO Registry

**DOI:** 10.1161/CIRCINTERVENTIONS.122.012780

**Published:** 2023-06-01

**Authors:** Stefano Benenati, Federico Giacobbe, Antonio Zingarelli, Fernando Macaya, Carloalberto Biolè, Angelica Rossi, Marco Pavani, Giorgio Quadri, Umberto Barbero, Andrea Erriquez, Tiziana Aranzulla, Chiara Cavallino, Dario Buccheri, Cristina Rolfo, Giuseppe Patti, Nieves Gonzalo, Alessandra Chinaglia, Giuseppe Musumeci, Javier Escaned, Ferdinando Varbella, Enrico Cerrato, Italo Porto

**Affiliations:** 1 Cardiovascular Disease Chair, Department of Internal Medicine (Di.M.I.); 2 University of Genoa, Italy (S.B., I.P.).; 3 Cardiology Department, AOU Citta` della Salute e della Scienza di Torino, Turin, Italy (F.G.).; 4 Cardiovascular Disease Unit, IRCCS Ospedale Policlinico San Martino, IRCCS Italian Cardiology Network, Genova, Italy (A.Z., I.P.).; 5 Hospital Clínico San Carlos, IdiSSC, Universidad Complutense de Madrid, Spain (F.M., N.G., J.E.).; 6 Division of Cardiology, San Luigi Gonzaga University Hospital, Orbassano, Turin, Italy (C.B., A.C.).; 7 Division of Cardiology, Azienda Ospedaliera Brotzu, Cagliari, Italy (A.R.).; 8 Interventional Cardiology Unit, San Luigi Gonzaga University Hospital, Orbassano and Rivoli Infermi Hospital, Italy (M.P., C.R., G.M., F.V., E.C.).; 9 Division of Cardiology, Ordine Ospedale Mauriziano Umberto I, Torino (TO), Italy (G.Q., T.A.).; 10 Division of Cardiology Ospedale Maggiore Ss. Annunziata - Savigliano (CN), Italy (U.B.).; 11 Division of Cardiology, University of Ferrara, Italy (A.E.).; 12 Division of Cardiology, Sant’Andrea Hospital, Vercelli, Italy (C.C.).; 13 Interventional Cardiology Unit, Department of Cardiology, S. Antonio Abate Hospital, Trapani, Italy (D.B.).; 14 University of Eastern Piedmont, Department of Thoracic and Cardiovascular Diseases, Maggiore della Carità Hospital, Novara, Italy (G.P.).

**Keywords:** coronary artery dissection, spontaneous, myocardial infarction, percutaneous coronary intervention, stents

## Abstract

**Background:**

The optimal management of patients with spontaneous coronary artery dissection remains debated.

**Methods:**

Patients enrolled in the DISCO (Dissezioni Spontanee Coronariche) Registry up to December 2020 were included. The primary end point was major adverse cardiovascular events, a composite of all-cause death, nonfatal myocardial infarction, and repeat percutaneous coronary intervention (PCI). Independent predictors of PCI and medical management were investigated.

**Results:**

Among 369 patients, 129 (35%) underwent PCI, whereas 240 (65%) were medically managed. ST-segment–elevation myocardial infarction (68% versus 35%, *P*<0.001), resuscitated cardiac arrest (9% versus 3%, *P*<0.001), proximal coronary segment involvement (32% versus 7%, *P*<0.001), and Thrombolysis in Myocardial Infarction flow 0 to 1 (54% versus 20%, *P*<0.001) were more frequent in the PCI arm. In-hospital event rates were similar. Between patients treated with PCI and medical therapy, there were no differences in terms of major adverse cardiovascular events at 2 years (13.9% versus 11.7%, *P*=0.467), all-cause death (0.7% versus 0.4%, *P*=0.652), myocardial infarction (9.3% versus 8.3%, *P*=0.921) and repeat PCI (12.4% versus 8.7%, *P*=0.229). ST-segment–elevation myocardial infarction at presentation (odds ratio [OR], 3.30 [95% CI, 1.56–7.12]; *P*=0.002), proximal coronary segment involvement (OR, 5.43 [95% CI, 1.98–16.45]; *P*=0.002), Thrombolysis in Myocardial Infarction flow grade 0 to 1 and 2 (respectively, OR, 3.22 [95% CI, 1.08–9.96]; *P*=0.038; and OR, 3.98 [95% CI, 1.38–11.80]; *P*=0.009) and luminal narrowing (OR per 5% increase, 1.13 [95% CI, 1.01–1.28]; *P*=0.037) were predictors of PCI, whereas the 2B-angiographic subtype predicted medical management (OR, 0.25 [95% CI, 0.07–0.83]; *P*=0.026).

**Conclusions:**

Clinical presentation and procedural variables drive the choice of the initial therapeutic approach in spontaneous coronary artery dissection. If PCI is needed, it seems to be associated with a similar risk of short-to-mid-term adverse events compared to medical treatment.

**Registration:**

URL: https://www.clinicaltrials.gov; Unique identifier: NCT04415762.

**What is Known**

- Contemporary guidelines and expert consensus support medical treatment in most spontaneous coronary artery dissection cases, limiting percutaneous coronary intervention to high-risk anatomies and hemodynamically unstable patients. Unfortunately, percutaneous coronary intervention in patients with spontaneous coronary artery dissection is burdened by a high risk of complications and its benefit is still debated.
- Data comparing the clinical outcomes of patients treated invasively or medically are scant and discordant.

**What The Study Adds**

- Notwithstanding the higher risk profile of the population undergoing percutaneous coronary intervention, the occurrence of short-to-mid-term major adverse events in spontaneous coronary artery dissection patients treated with an initial invasive or conservative approach was similar.
- ST-segment–elevation myocardial infarction at presentation, proximal coronary segment location, increased luminal narrowing, and suboptimal Thrombolysis in Myocardial Infarction flow increased the likelihood of undergoing percutaneous coronary intervention.
- 2B spontaneous coronary artery dissection subtype was the only predictor of an initial conservative management.

Spontaneous coronary artery dissection (SCAD) is defined as a coronary epicardial dissection not related to trauma, iatrogenic injuries, or atherosclerotic disease. It accounts for up to 4% of acute coronary syndromes in the general population.^1–3^ Although current guidelines suggest pursuing a conservative strategy whenever clinically possible,^1,4^ uncertainty remains regarding the potential advantage of performing percutaneous coronary intervention (PCI) as first approach to avoid early progression and propagation of hematoma to proximal segments, with a consequent risk of serious adverse events. Additionally, in many cases of ongoing ischemia or hemodynamic instability, a conservative approach is arguable and ineffective in clinical practice.^5^ However, PCI is burdened by higher procedural risks and lower success rate in SCAD compared to classical atherosclerotic disease.^4^ Furthermore, spontaneous healing is often possible in the short-to-mid-term,^6^ whereas bailout revascularization is rare in conservatively managed individuals.^4,7,8^

In the absence of randomized trials, the choice between these approaches remains largely based on expert recommendations^1,4^ and observational evidence, with few studies^9,10^ and meta-analyses^11,12^ specifically comparing an initial strategy of invasive versus conservative management.

In a SCAD cohort enrolled in the multicenter DISCO (Dissezioni Spontanee Coronariche) registry, we aimed to investigate: (1) the prevalence of PCI or medical therapy as the initial approach, (2) the predictors of the initial treatment strategy, and (3) the association between the initial treatment strategy and clinical outcomes.

## Methods

Details on study protocol and procedures, as well as end points definitions, have been previously reported.^13,14^ Briefly, the DISCO IT/SPA (Dissezioni Spontanee Coronariche Italy/Spain) is a multicenter, observational registry in which patients with SCAD are retrospectively enrolled across 26 Italian and Spanish centers. The study was approved by the institutional review boards. An informed consent to be submitted to the patient and information leaflets for general practitioners were drawn up. All the data and methods will be made available to third parties available upon reasonable request to the corresponding author.

Demographic data, clinical presentation, treatment modality, angiographic findings, administered drugs, early and late outcomes were extracted by the hospital computer databases of each center. In case of missing data, the General Practitioner or the patient himself was contacted or an in-person visit was scheduled to obtain the necessary information. A dedicated electronic case report form was designed on Cardiogroup.org research website to aid data collection. A detailed description of any requested variable was provided to avoid misunderstandings. A specific section of the electronic case report form was designed to collect adverse events. These were adjudicated by the coordinating center after revision of coronary angiographies and available clinical information (clinical presentation, ECG, troponin values). A dedicated data manager (L.L.S.) was in charge of source verification, quality control, and queries generation to minimize bias. All coronary angiograms were reviewed by a core laboratory involving the coordinating center (Rivoli Infermi Hospital and San Luigi Gonzaga Hospital, Turin, Italy) and the leading recruiting center of the study (Hospital Clinico San Carlos, Madrid, Spain). Angiographies were first sent to the coordinating center, where 2 experienced interventional cardiologists (G.Q., C.R.) reviewed the case and confirmed the diagnosis of SCAD. In this phase, the cardiologists were blinded to clinical data and angiogram time points. In case of disagreement, consensus was achieved after discussion with a third interventional cardiologist (F.T.). Additional angiograms performed during index hospitalization or follow-up as well as any intracoronary imaging available were also reviewed to improve the diagnostic accuracy. Angiographic classification was performed in a second stage by both the Italian (G.Q., C.R., E.C.) and the Spanish center (R.M., F.M., J.E.). Two online meetings were held by core lab members to reach a consensus on the specific criteria to be considered for classification. Another online meeting was held to discuss unclear cases to optimize the diagnostic process.

The primary outcome was the rate of major adverse cardiovascular events (MACE), a composite of all-cause death, nonfatal myocardial infarction (MI), and any unplanned PCI. Individual components of the primary outcome and in-hospital bleeding, classified according to the Bleeding Academic Research Consortium definition,^15^ were also assessed. MI was defined according to the fourth universal definition.^16^ Follow-up was performed through telephone call and/or electronical medical record review.

For the present analysis, patients enrolled between January 2009 and December 2020 were included and grouped according to the initial treatment strategy (ie, the approach chosen at diagnosis confirmation after coronary angiogram) in conservatively managed (medical therapy) or immediate PCI. Patients undergoing coronary artery bypass graft were excluded. SCAD was angiographically classified according to published criteria.^4,17^ Proximal coronary location was defined as the involvement of left main or the proximal segments of left anterior descending, left circumflex, or right coronary artery. Procedural success was intended as the ability to cross the SCAD lesion(s) followed by Thrombolysis in Myocardial Infarction (TIMI) flow improvement by at least 2 grades from the baseline after balloon inflation or stent placement. Spontaneous healing was defined as SCAD resolution, that is, absence of contrast staining with residual stenosis <50%, at follow-up imaging (either computerized tomography or coronary angiogram).^7^

### Statistical Analysis

Categorical variables were reported as frequencies and percentages and compared by means of the χ^2^ or Fisher test, as appropriate. Continuous variables were visually assessed for distribution and subsequently reported as mean (SD) or median (quartile 1–quartile 3 [Q1–Q3]) and compared through the Student *t* test or Wilcoxon-Mann-Whitney *U* test, as appropriate.

Binary logistic regressions were run to obtain the predictors of invasive management. To adjust for covariates, significant univariate predictors were entered in a multivariable model. Odds ratios (OR) and associated 95% CIs were derived.

The cumulative unadjusted frequencies of MACE and their individual components in the 2 groups were obtained with the Kaplan-Meier method and compared through the log-rank test. Clinical events were censored at 2 years. Two-tailed *P*<0.05 were considered statistically significant. The analysis was conducted using R (The R Foundation for statistical computing, Vienna, version 3.6.2).

## Results

### Demographic, Clinical, and Procedural Features

**Figure 1.**
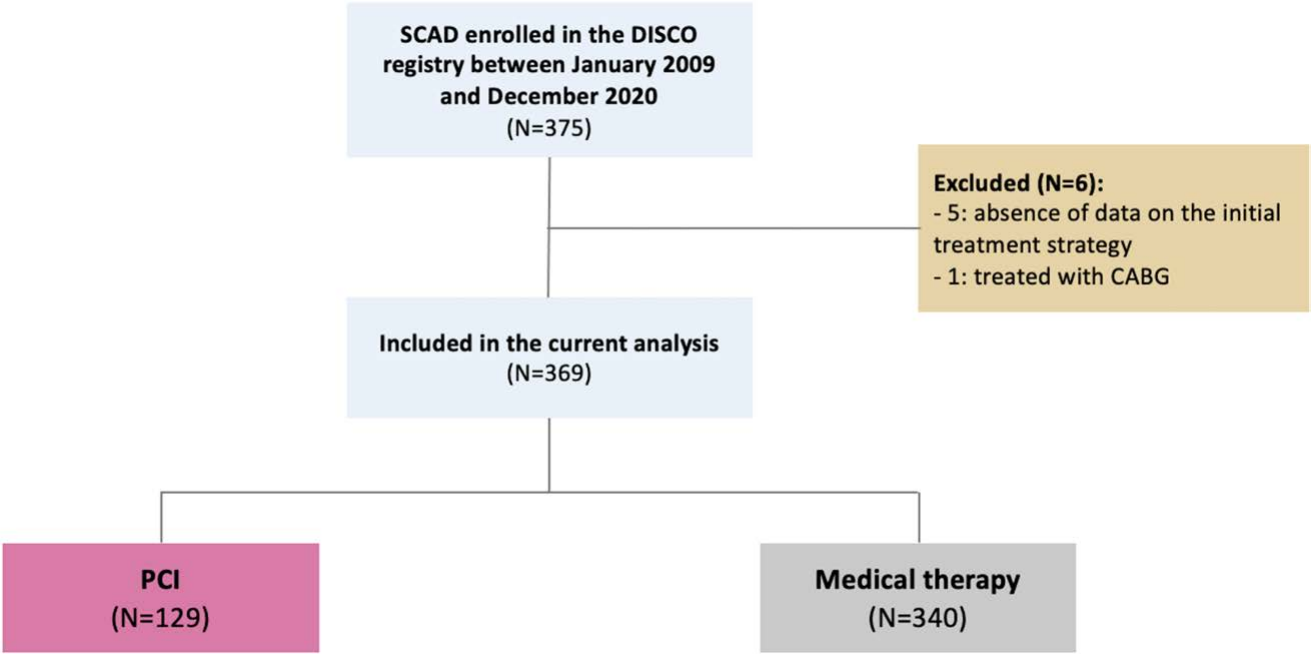
**Study flow.** CABG indicates coronary artery bypass graft; DISCO, Dissezioni Spontanee Coronariche; PCI, percutaneous coronary intervention; and SCAD, spontaneous coronary artery dissection.

Figure 1 displays the study flow. The population enrolled up to December 2020 encompassed 375 patients. Among them, 5 patients with unspecified first treatment approach and one patient who underwent coronary artery bypass graft were excluded. Thereby, out of a total of 369 patients, 129 (35%) were immediately treated with PCI, and 240 (65%) were conservatively managed after the angiographic diagnosis.

Baseline clinical characteristics are summarized in Table 1. Mean age was 51±11 years in the PCI arm and 53±10 years in the conservative group. In both arms, most patients were female (84% versus 87%, *P*=0.466). Patients undergoing PCI were less frequently affected by hypertension (26% versus 41%, *P*=0.007). In the same arm, ST-segment–elevation myocardial infarction (68% versus 35%, *P*<0.001) and resuscitated cardiac arrest (9% versus 3%, *P*=0.032) were more frequent. A higher prevalence of patients with reduced ejection fraction (66% versus 42%, *P*<0.001) and a longer hospital stay was also noted (7 [6–10] versus 6 [5–8] days, *P*=0.001). Only 6% of patients undergoing revascularization were discharged on single antiplatelet therapy, as opposed to a third (33%) of those who were managed conservatively.

**Table 1.**
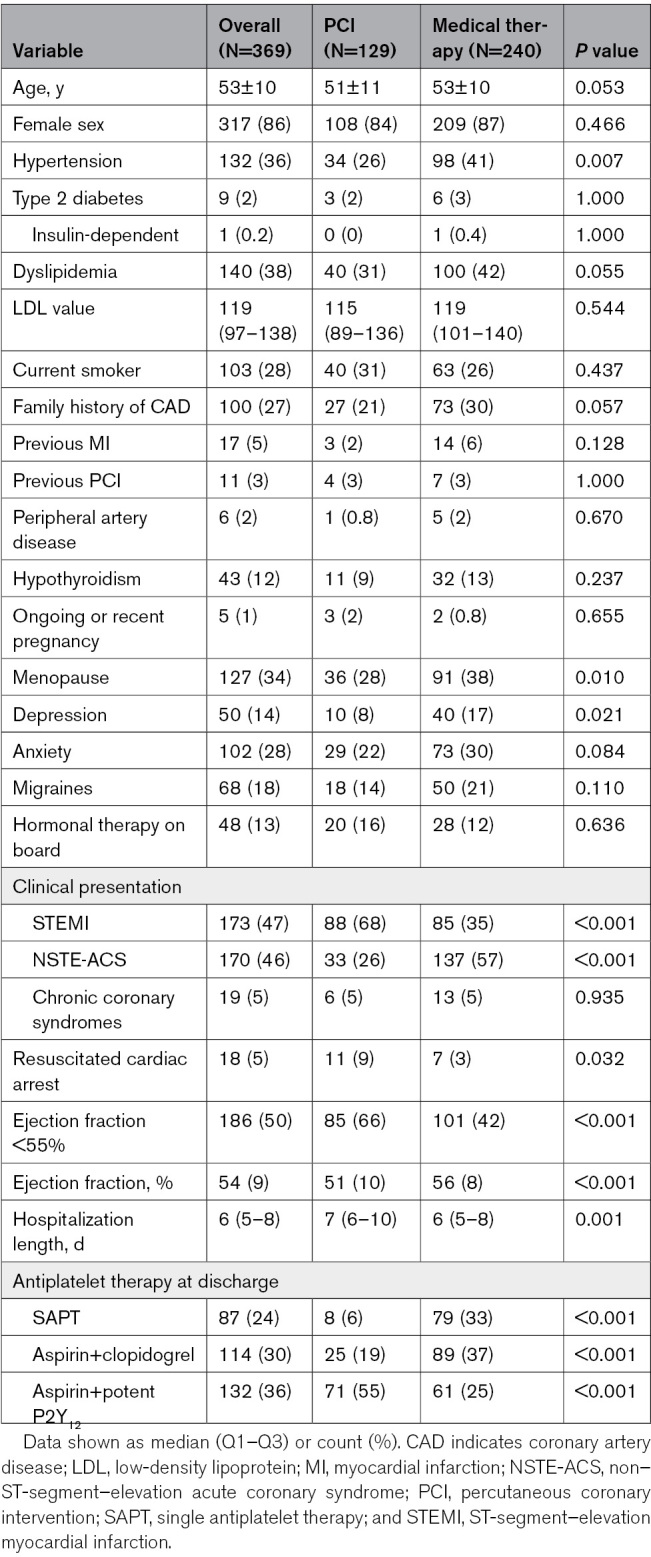
Baseline Characteristics

Table 2 reports the procedural features. Over 70% of patients underwent coronary angiography through the radial route, with bailout femoral access being more frequent in those who had undergone PCI (9% versus 1.2%, *P*<0.001). Proximal coronary segment location (32% versus 7%, *P*<0.001), TIMI flow grade 0 to 1 (54% versus 20%, *P*<0.001), and multivessel SCAD (19% versus 9%, *P*=0.015) were more frequent among patients treated with PCI, likewise left main involvement (6% versus 0.4%, *P*=0.001). There was also a significant difference in terms of diameter luminal narrowing (90±17% versus 82±18%, *P*<0.001) and angiographic types prevalence between the groups (22% versus 13% [*P*=0.030] for type 1; 17% versus 30% [*P*=0.011] for type 2A; 9% versus 30% [*P*<0.001] for type 2B; 9% versus 5% [*P*=0.316] for type 3; and 40% versus 20% [*P*<0.001] for type 4). Intracoronary imaging, and particularly intravascular ultrasound, was predominantly used in revascularized patients (29% versus 3%, *P*<0.001). In particular, among type 4 patients with SCAD, 53% underwent PCI and, of these, 33% received intracoronary imaging. Procedural success was reached in the 77% of patients treated with PCI.

**Table 2.**
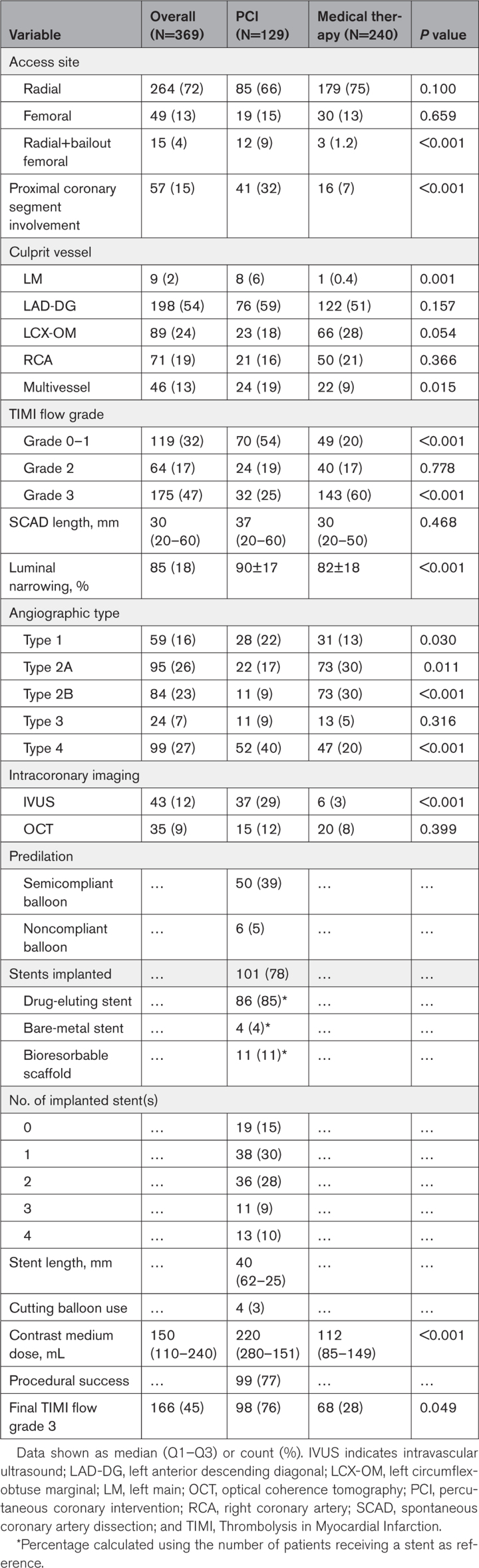
Procedural Characteristics

### Predictors of Invasive Management

Logistic regression results are reported in Table 3. At multivariable analysis, ST-segment–elevation myocardial infarction at presentation (OR, 3.30 [95% CI, 1.56–7.12]; *P*=0.002), proximal coronary segment involvement (OR, 5.43 [95% CI, 1.98–16.45]; *P*=0.002), TIMI flow grade 0-1 and 2 (respectively: OR, 3.22 [95% CI, 1.08–9.96]; *P*=0.038; and OR, 3.98 [95% CI, 1.38–11.80]; *P*=0.009), and the severity of diameter luminal narrowing (per 5% increase, OR, 1.13 [95% CI, 1.01–1.28]; *P*=0.037) were all associated with increased probability of PCI. On the contrary, angiographic subtype 2B independently predicted a conservative approach (OR, 0.25 [95% CI, 0.07–0.83]; *P*=0.026).

**Table 3.**
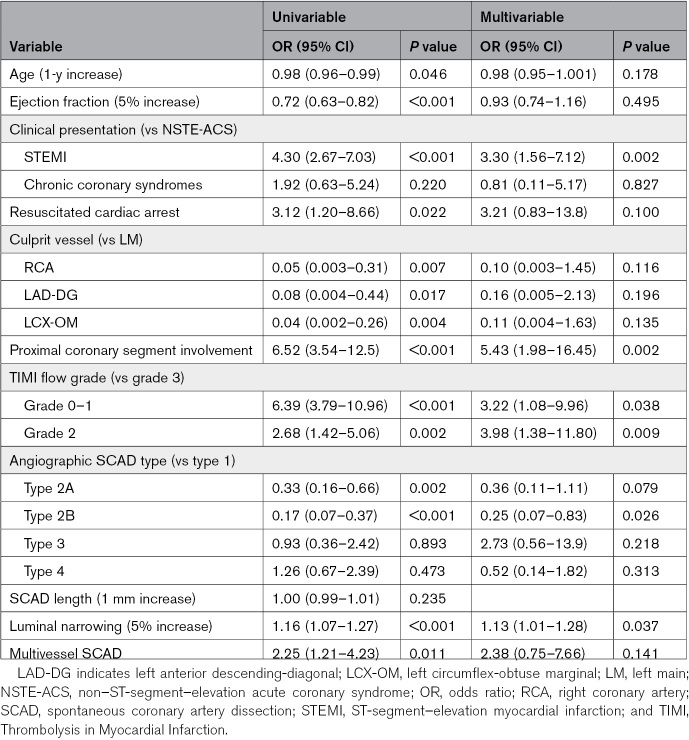
Univariate and Multivariate Logistic Regression Analyses for Invasive Management

### In-Hospital Clinical Outcomes

In-hospital MACE occurred in 6.9% and 8.3% of patients in the PCI and medical therapy groups, respectively (*P*=0.795). In-hospital death, myocardial infarction, and repeat PCI rates were similar (0.7% versus 0.4%, *P*=0.532; 4.7% versus 5.8%, *P*=1; and 6.2% versus 6.7%, *P*=0.956, respectively). Bleeding occurred in 3.9% versus 0.8% of patients, with a trend toward statistical significance (*P*=0.053). With the exception of one moderate (Bleeding Academic Research Consortium type 2) event registered in the medical therapy arm, all bleeding events were trivial. Table 4 summarizes the events.

**Table 4.**
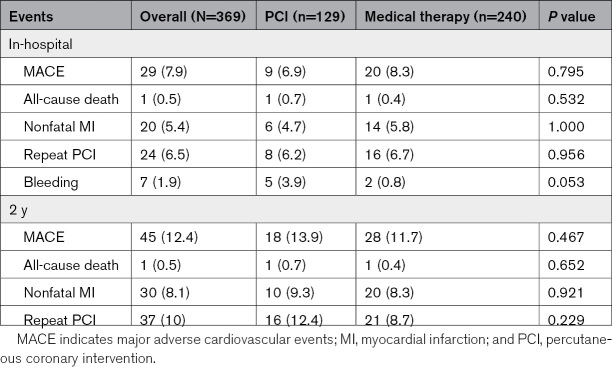
Clinical Events

### Angiographic Follow-Up and Clinical Outcomes Up to 2 Years

Overall, 36% of patients underwent angiographic follow-up within a median time of 83 (8–262) days. Among medically treated patients who had undergone imaging at follow-up, the rate of SCAD healing was 56%. As previous studies have shown spontaneous healing to be infrequent before 3 months, we stratified the population according to this threshold. In patients undergoing imaging follow-up before 3 months, the median time to imaging was 8 (5–51) days. In this group, the rate of healing was 34%. In the counterpart undergoing imaging follow-up ≥3 months, the median time to imaging was 270 (177–439) days. In this group, the rate of spontaneous healing reached 80%.

**Figure 2.**
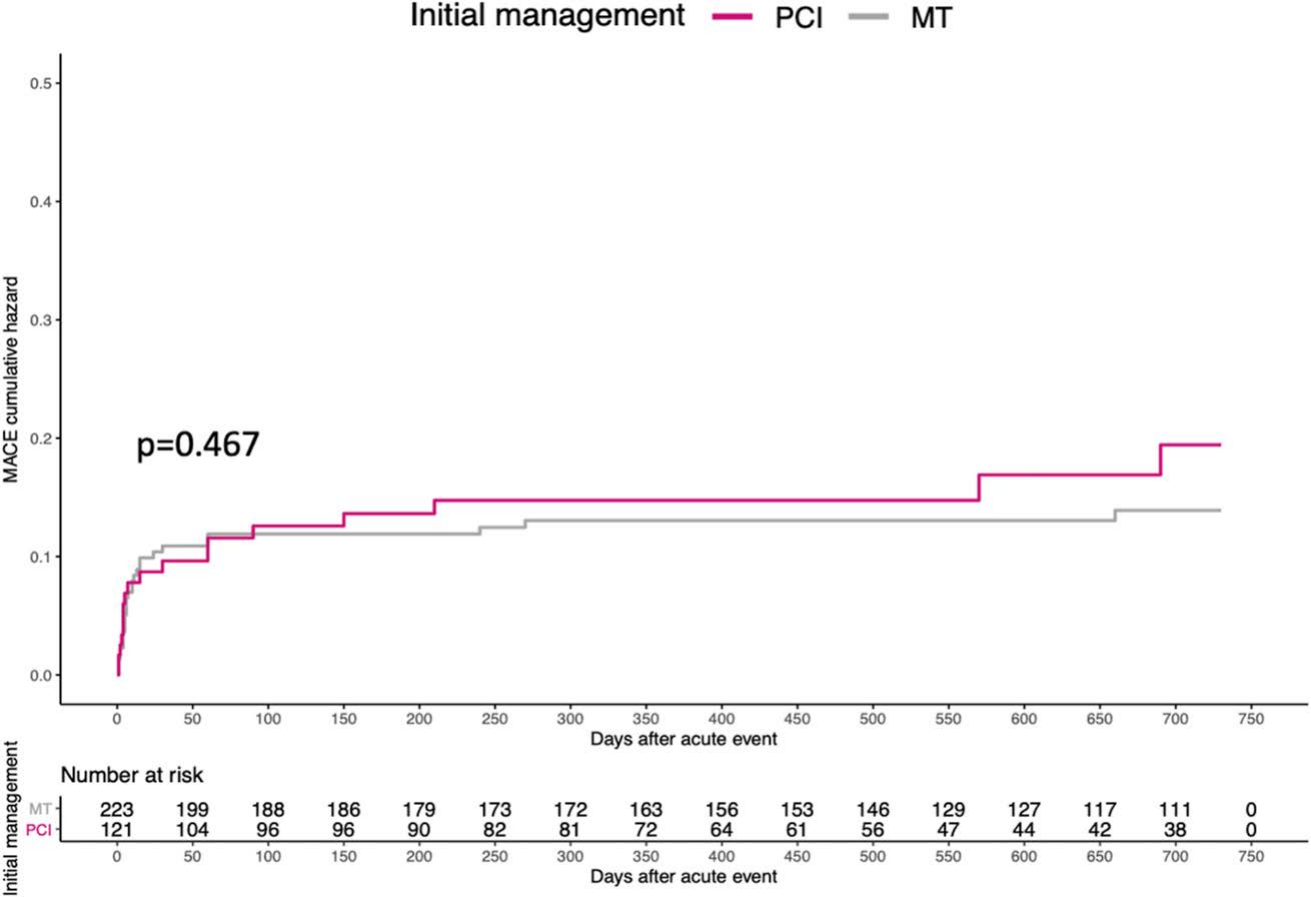
**Cumulative events curves for the composite end point of major adverse cardiovascular events (MACE).** MT indicates medical therapy; and PCI, percutaneous coronary intervention.

At 2 years, clinical data were available for 93% of patients, with a median follow-up duration of 18 (8–24) months. Events frequencies are shown in Table 4 and the cumulative incidence of MACE in Figure 2. The primary end point occurred in 13.9% of patients who had initially undergone PCI and in 11.7% of those initially treated with medical therapy (*P*=0.467). All-cause death occurred in 0.7% and 0.4% of patients, respectively (*P*=0.652); nonfatal MI was registered in 9.3% versus 8.3% of patients, respectively (*P*=0.921); whereas PCI was repeated in 12.4% versus 8.7% of patients, respectively (*P*=0.229).

## Discussion

In the DISCO cohort, most patients were conservatively managed, yet PCI was eventually adopted in around one-third of them. ST-segment–elevation myocardial infarction at presentation, the involvement of a proximal coronary segment, a suboptimal TIMI flow in the culprit vessel at first coronary angiogram, and an increasing severity of luminal narrowing independently predicted the choice of PCI as initial strategy, whereas the 2B-angiographic subtype was the only predictor of medical management. No significant differences were detected in terms of in-hospital and mid-term outcomes.

SCAD is increasingly recognized as an important cause of cardiovascular morbidity and mortality, particularly in young females.^1,18^ Unlike atherothrombosis, SCAD is presumed to result from the development of a parietal hematoma within the tunica media or, less frequently, between the intima and the media, leading to *ab extrinseco* lumen obstruction.^4^ Based on this pathophysiology, key differences with atherosclerotic disease exist from an interventional standpoint. First, as intramural hematoma does not always communicate with the true arterial lumen, inside-out wall compression during coronary balloon inflation or stent placement potentially results in hematoma squeezing, leading to SCAD propagation. Moreover, the risk of iatrogenic dissection in patients with SCAD is higher than usual, especially as a result of aggressive guiding catheter manipulation and deep coronary intubation.^19^ Further drawbacks of coronary intervention include accidental wiring of the false lumen and stent malapposition due to late hematoma resorption.^20^ Thereby, coronary revascularization, and particularly PCI, is associated with high procedural risks and increased frequency of failure in SCAD.^21,22^ On this basis, caution is advocated by expert consensus documents, which favor a conservative approach when there is no hemodynamical instability, ongoing ischemia, or compromised epicardial flow.^1,4^ Noteworthy, most SCAD heal spontaneously over a short-to-mid-term follow-up.^6,9,23^ Despite accumulating studies,^7,24–27^ only few authors have specifically investigated the distinctive features of revascularized patients with SCAD, as well as their clinical outcomes, as compared to those who are medically treated.^9,10,26^

Our analysis expands the current knowledge by investigating a larger cohort derived from several Italian and Spanish centers. Among revascularized patients, we restricted the analysis to those treated with PCI, by far the most adopted revascularization strategy at present. Although we confirmed the overall trend toward a conservative approach, the proportion of patients treated with PCI was higher than several previous studies,^25,28–30^ which, at least in part, might have resulted from the lack of a standardized treatment protocol across the participating centers. In agreement with the existing evidence, revascularized patients were those perceived at higher risk. However, it is not surprising that the angiographic subtype 2B portended a conservative management, as, in this SCAD phenotype, the hematoma extends toward the far periphery of the coronary tree,^17^ discouraging an interventional approach.

In line with previous reports^7–9,26,31^ procedural success was as high as 75% in our immediate PCI arm, and, as opposed to another cohort study,^9^ there was no need for emergency coronary artery bypass graft as a result of procedural failure, suggesting that PCI may be effectively performed in appropriately selected patients. We did not appreciate significant differences in the rate of in-hospital adverse events between the treatment strategies. In this regard, differently from the previous literature, we also provide an estimation of hemorrhagic events, which were rare and all nonmajor. We also found substantial equipoise between the strategies with respect to clinical outcomes. Notably, the frequency of all-cause mortality was very low in both arms (<1% at 2-year follow-up), and all deaths occurred during hospital stay. Therefore, in case the clinical judgment favors an interventional approach, PCI does not seem to expose patients with SCAD to an excess of adverse events, even if the interventional strategy is adopted in those who are deemed, at baseline, at higher risk. Notably, this finding is in contrast with previous meta-analyses suggesting heightened risk of target vessel revascularization in patients treated with an interventional approach.^11,12^ With regard to the abovementioned studies, however, it should be noted that the disadvantage of PCI was primarily driven by older reports,^9,26^ in which contemporary revascularization techniques were likely unavailable or poorly implemented.

Despite angiographic follow-up being performed in only one patient in 3, complete SCAD healing was rarer compared to previous studies.^6,9,23,25^ However, after stratifying patients according to the time of follow-up, we found a rate of 80% in those who had undergone imaging after 3 months. This is consistent with the fact that spontaneous healing is very uncommon within the first month,^28^ whereas its frequency increases from 3 months onwards.^6,9,23^ Many re-evaluations performed in our retrospective series were in-hospital, and those carried out beyond the first trimester were the minority. Additionally, although most angiographic follow-up were planned, a certain amount was driven by clinical instability, which intuitively heightens the likelihood of SCAD persistence or worsening. Furthermore, in a seminal work by Tweet et al,^9^ the rate of spontaneous healing was 73% even at a median of 2.4 (0.9–6.2) years.

### Limitations and Strengths

This analysis is limited by its retrospective and observational design. We cannot exclude selection bias as well as the influence of potential confounders. Furthermore, some key information might have been not collected, and the accuracy of the analysis might be affected by missing values. However, it should be also acknowledged that observational studies represent the main source of knowledge in the rare and complex field of SCAD,^32^ as conducting adequately-powered randomized studies will always be challenging. Although our study represents one of the largest SCAD cohorts studied so far, the absolute number of patients remains low, which, together with the low incidence of adverse events, significantly limits the statistical power, affecting both the comparison of clinical events rates and the multivariable analysis. For these reasons, our findings should be interpreted as hypothesis-generating. MACE (death, MI, target lesion revascularization) might not reflect some of the technical issues derived from complex interventions in patients with SCAD (such as loss of side branches, inadequate final TIMI flow, larger MI). Type 4 SCAD represents a scenario where SCAD diagnosis and treatment can be even more challenging than other subtypes. Although we do not provide specific analyses in this regard, a subanalysis of the DISCO registry has been recently published.^33^

Finally, intravascular imaging use was not as high as in other series.^23,27,34,35^ However, it is in line with some seminal articles in the field.^9,36^ It should be noted that, despite being of undoubtful help for SCAD diagnosis, intravascular imaging still remains an ancillary technique, as diagnosis and classification of SCAD are in fact based on angiographic appearance according to expert consensus documents.^1,4^ Furthermore, the central adjudication of angiographic images guarantees the appropriateness of SCAD diagnosis.

### Conclusions

The choice between medical or interventional management of SCAD is driven by clinical presentation and procedural aspects, including stenosis severity, the angiographic type of SCAD, and the involvement of proximal coronary segments. In this DISCO cohort subanalysis, the primary treatment approach was not associated with different short-to-mid-term adverse events, although the limited sample size and the observational design necessarily make these results hypothesis-generating.

## Article Information

### Source of Funding

None.

### Disclosures

Dr Porto reports consultant or speaker fees from Biotronik, ABIOMED, Terumo, Philips, Sanofi, Amgen, Daiichi-Sankyo, Astra Zeneca, Bayer, and PIAM, not related to this work. The other authors report no conflicts.

## Appendix

DISCO Contributors: Matteo Bianco, Gianmarco Annibali, Francesco Bruno, Fabrizio D’Ascenzo, Annamaria Nicolino, Chiara Bernelli, Pablo Salinas, Luca Bettari, Elisabetta Bordoni, Vincenzo Infantino, Alfonso Gambino, Andrea Rognoni, Marco Mennuni, Sebastian Cinconze, Alberto Boi, Francesco Tomassini, Alfonso Franzè, Luca Lo Savio, Bruno Loi, Mario Iannaccone, Michele De Benedictis. Gianluca Campo, Fabrizio Ugo, Alessandra Truffa Giachet, Giuseppe Pietro Greco Lucchina, Francesco Cassano, Andrea Gagnor, Federico Beqaraj, Luca Gaido, Matteo Perfetti, Primiano Lombardi.
